# Prevention of mother-to-child transmission of HIV in rural Uganda: Modelling effectiveness and impact of scaling-up PMTCT services

**DOI:** 10.3402/gha.v8.26308

**Published:** 2015-02-27

**Authors:** Elin C. Larsson, Anna Mia Ekström, George Pariyo, Göran Tomson, Mohammad Sarowar, Rose Baluka, Edward Galiwango, Anna Ekéus Thorson

**Affiliations:** 1 Department of Public Health Sciences Global Health/IHCAR, Karolinska Institutet, Stockholm, Sweden; 2 Department of Women’s and Children’s Health, Karolinska Institutet, Stockholm, Sweden; 3 Department of Infectious Diseases, Karolinska University Hospital, Stockholm, Sweden; 4 Deptartment of Health Policy, Planning and Management, Makerere University School of Public Health, Kampala, Uganda; 5 Iganga-Mayuge Health and Demographic Surveillance Site, Iganga, Uganda; 6 Department of Learning, Informatics, Management and Ethics (MMC), Karolinska Institutet, Stockholm, Sweden

**Keywords:** HIV, effectiveness, prevention of mother-to-child transmission, population-based, cohort

## Abstract

**Background:**

The reported coverage of any antiretroviral (ARV) prophylaxis for prevention of mother-to-child transmission (PMTCT) has increased in sub-Saharan Africa in recent years, but was still only 60% in 2010. However, the coverage estimate is subject to overestimations since it only considers enrolment and not completion of the PMTCT programme. The PMTCT programme is complex as it builds on a cascade of sequential interventions that should take place to reduce mother-to-child transmission (MTCT) of HIV: starting with antenatal care (ANC), HIV testing, and ARVs for the woman and the baby.

**Objective:**

The objective was to estimate the number of children infected with HIV in a district population, using empirical data on uptake of PMTCT components combined with data on MTCT rates.

**Design:**

This study is based on a population-based cohort of pregnant women recruited in the Iganga-Mayuge Health and Demographic Surveillance Site in rural Uganda 2008–2010. We later modelled different scenarios assuming increased uptake of specific PMTCT components to estimate the impact on MTCT for each scenario.

**Results:**

In this setting, HIV infections in children could be reduced by 28% by increasing HIV testing capacity at health facilities to ensure 100% testing among women seeking ANC. Providing ART to all women who received ARV prophylaxis would give an 18% MTCT reduction.

**Conclusions:**

Our results highlight the urgency in scaling-up universal access to HIV testing at all ANC facilities, and the potential gains of early enrolment of all pregnant women on antiretroviral treatment for PMTCT. Further, to determine the effectiveness of PMTCT programmes in different settings, it is crucial to analyse at what stages of the PMTCT cascade that dropouts occur to target interventions accordingly.

The reported coverage of antiretroviral (ARV) prophylactic therapy for prevention of mother-to-child transmission (PMTCT) of HIV has increased in sub-Saharan Africa in recent years, but was still limited to 60% in 2010 (1). The estimated coverage of PMTCT services in many low-income countries are subject to overestimations since it only considers enrolment into the programme, and does not include whether the individual PMTCT components are fulfilled or not (2). The PMTCT programme is complex since it builds on sequential interventions that should take place to reduce mother-to-child transmission (MTCT) of HIV: starting with antenatal care (ANC), HIV testing, antiretroviral drugs for the pregnant woman and postpartum to the infant, as well as continued antiretroviral treatment (ART) to the woman within the frame of the WHO option B (ART throughout the breastfeeding period) or B+ (ART for life) regimens (2). Without any intervention, the risk of MTCT is 35–40% but could be brought down to around 1% in high-income settings with optimal resources available, and has been reported at 5% in trial settings in low- and middle-income countries (3–5) . However, health systems’ shortcomings, such as poor referral systems for HIV testing, inadequate PMTCT counselling, and dropouts from the programme, hamper the effectiveness of PMTCT services and lead to HIV infections among children that could have been prevented (6–8) . Barker et al. have modelled that if assuming an uptake of 80% in the following PMTCT programme components: ANC access, HIV- and CD4 testing, and ARV prophylaxis, it would translate into a situation where the final uptake of ARV prophylaxis among women in need would be 51% (7).

In Uganda, the estimated proportion of HIV-infected pregnant women that receive any kind of ARVs for PMTCT has increased from 34% in 2007 to 53% in 2009 (2, 9). The recommended ARV prophylactic regimens at the time of study were based on the WHO 2006 recommendations and were for the mother either dual ARVs with zidovudine (AZT) and lamivudine up to 1 week after delivery, or single dose nevirapine (sd-NVP). Infants received AZT for 1 week or sd-NVP. ART was provided only to those women who were eligible for treatment for their own health, that is, those who were either in clinical stage III or IV or those who had CD4 cell count levels below 250 mm^3^ at the time of study (10).

Most estimates, as well as research on coverage, and effectiveness of PMTCT programmes, use national, aggregated data, or are based on health-facility based studies (7, 11–13) . This study uses population-based data from a cohort of pregnant women recruited in a Health and Demographic Surveillance Site (HDSS) in rural Uganda. The aim of the study was to estimate the proportion of children infected with HIV at birth, and at 6 months postpartum in a district population, by using recent empirical cohort data on uptake of PMTCT programme components combined with the data available on MTCT rates. Further, the study aim was to identify what PMTCT interventions that would have highest impact on MTCT in this setting.

## Methods

### Study setting and data collection

The empirical data on PMTCT programme coverage derives from a population-based cohort study of pregnant women carried out between 2008 and 2010 in the Iganga-Mayuge HDSS in rural Uganda. The data collection process has been described in detail previously (14). In brief, in an HDSS, a defined population is followed over time to produce population-based data in a context that normally lacks reliable population- and health data. The regular field assistants in the HDSS collected the data for this study, and entered this into the HDSS database. The first author in collaboration with HDSS staff trained the field assistants.

Data were collected from households and health facilities on ANC attendance, HIV testing, ARV prophylaxis, and type of ARV prophylaxis provided to the mother and mode of infant feeding. Dropout at each step was analysed.

The Iganga-Mayuge HDSS population consists of an estimated 68,000 individuals living in 12,000 households. They are followed-up regularly and data on vital events such as births, deaths, and migration are collected. The HDSS area is predominantly rural (80%), apart from Iganga town which is semi-urban, and the main source of income in the area is subsistence farming. The HIV prevalence in 2011 (in persons aged 15–49) in East-Central Uganda is estimated at 5.8% (15). ANC is available at health facilities at all levels with an estimated ANC attendance (at least one visit) of 96% in this setting (14). At the time of study, about half of the health facilities also provided HIV testing services as part of ANC, and one third was accredited to provide ARV prophylaxis for PMTCT. According to the national PMTCT guidelines, women who visited health facilities for ANC that did not have HIV testing services should be referred to neighbouring health facilities for testing. The recommended ARV prophylactic regimens at the time of study were based on the WHO 2006 recommendations as explained in the introduction.

### Base case HDSS cohort study

**Fig. 1:**
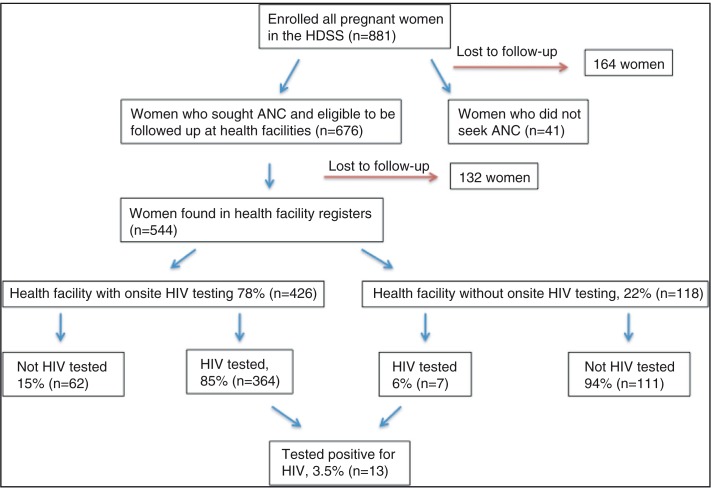
Iganga-Mayuge Health and Demographic Surveillance Site – cohort of pregnant women.

During the regular HDSS data collection in May–July 2008, all households were screened for pregnant women and all of these women (*n*=881) were followed throughout their pregnancy and 6 months after delivery (Fig. 1). In addition, record reviews were performed at the health facilities where the women in the cohort reported that they had sought ANC. Data on PMTCT dropout among HIV-infected women (*n*=13) were collected from the ANC records for everyone, and through semi-structured interviews for about half of them (*n*=7). Only seven of the 13 HIV-infected women could be interviewed since one was not found, two had moved out of the HDSS area, and two denied being HIV infected. The interviews were home-based, semi-structured using both closed and open-ended questions and conducted 12–20 months after delivery by a Ugandan midwife with long experience of PMTCT work.

### Decision tree analysis – base case PMTCT scenario

We performed a decision tree analysis with the main outcome being the estimated number of children infected with HIV at birth and at 6 months postpartum in the Iganga and Mayuge districts. In this analysis, the population-based data for the HDSS cohort were analysed using frequency tables to define the proportion of women covered by each stage of the PMTCT programme; this is referred to as the *base case PMTCT scenario*. Further, we modelled different scenarios assuming increased coverage of specific, individual PMTCT programme components to estimate the impact on paediatric HIV infections for each modelled scenario.

Given below is a summary of the base case PMTCT scenario:

1. ANC attendance 96%
2. Attending ANC at a facility with HIV testing services 78%
3. HIV tested (attending ANC at a facility with HIV testing services) 85%
4. HIV tested (attending ANC at a facility without HIV testing services) 6%
5. HIV prevalence 3.5%
6. HIV-infected women/baby pair receiving any type of ARV prophylaxis 83%
7. Women practicing safe feeding 0%

The details of the base case scenario are presented in Table 1. These data were then combined with the UNAIDS estimates of MTCT rates for different ARV prophylactic regimens, for different infant feeding practices, and for different CD4 cell count levels among the women (16). The combined data were subsequently entered as probabilities in a decision tree model created in Microsoft Excel and the model inputs are presented in Table 1. The HIV prevalence of 3.5% used in the analysis is based on the number of pregnant women in the HDSS cohort that were found to be HIV infected. Blood samples for CD4 cell count were not routine in the two districts; therefore, the number of HIV-infected children at 6 months is presented for both MTCT rates below and above 350 mm^3^ CD4 cell counts.

**Table 1:** Input values to the decision tree analysis input

Data from the HDSS cohort (base case) on probability of coverage for individual PMTCT programme components (*n*=717)	

Components	Probabilities
ANC attendance	0.96
Attending ANC at a facility with HIV testing services	0.78
HIV tested (attending ANC at a facility with HIV testing services)	0.85
HIV tested (attending ANC at a facility without HIV testing services)	0.06
HIV prevalence	0.035
HIV-positive women/baby pair receiving any ARV prophylaxis (*n*=13)	0.83
Mother ARV prophylaxis – sd-NVP^a^	0.33
Mother ARV prophylaxis – dual prophylaxis AZT^b^ and 3TC^c^	0.57
Mother ARV prophylaxis – ART (for her own health)	0.10
Women practicing safe feeding^d^	0
HIV mother-to-child transmission rates^e^	
Peripartum transmission rates for different ARV regimens	
No ARV prophylaxis	0.22
sd-NVP	0.12
Dual prophylaxis of AZT and 3TC	0.04
ART for her own health	0.02
Postpartum transmission rates per month of any breastfeeding (mixed or exclusive)	
Mother’s CD4 cell count ≤350 mm^3^	0.0157
Mother’s CD4 cell count >350 mm^3^	0.0051
If mother receives ART for her own health	0.002

a single dose nevirapine

b zidovudine

c lamivudine

d Safe feeding refers to exclusive breastfeeding or formula feeding

e Adopted from Ref. (16).

### Inference to Iganga and Mayuge district population

To estimate the number of HIV-infected children in the whole of the Iganga and Mayuge districts, we inferred the base case probabilities in Table 1 to the districts population of about 1,140,000 individuals in 2008 (16). Based on the Ugandan crude birth rate of 45/1,000 and the HIV prevalence of 3.5%, there was an expected 51,300 pregnant women in the two districts in 2008 of which 1,796 were estimated to be HIV infected.

### Estimated effectiveness for scenarios where an increase in the coverage of certain PMTCT components has been assumed

Different scenarios were modelled and the first modelling used the base case PMTCT coverage obtained from the HDSS cohort: ANC 96%, HIV testing 64%, and ARV prophylaxis 83%.

The other scenarios assumed an increase in coverage of the individual PMTCT programme components as compared to the base case scenario. This modelling was conducted to estimate how an increased coverage of *individual* PMTCT components would impact the estimated number of HIV-infected children at birth and at 6 months.

The effectiveness was estimated for the following four scenarios, using the base case PMTCT coverage but with the following assumed changes:

1. 100% ANC attendance
2. 100% uptake of HIV testing
3. Provision of ART throughout pregnancy and the breastfeeding period (corresponding to the current option B) to all HIV-infected women who received ARV prophylaxis (i.e. coverage 83%)
4. Optimal scenario, combining all of the above scenarios

To estimate the effectiveness of the base case PMTCT scenario as compared to a situation without any PMTCT services at all, a scenario that assumed that no one would receive any PMTCT service at all was also modelled.

Further, for the scenarios presented above the number of children infected with HIV at 6 months was calculated for two different situations; one with the mothers CD4 cell count levels set to >350 mm^3^ (transmission rate of 0.51%/month of breastfeeding) and one with CD4 cell count levels set to ≤350 mm^3^ (transmission rate of 1.57%/month of breastfeeding) (16).

To estimate the relative effect on the number of HIV-infected children for each scenario, all scenarios were compared to the estimated number of HIV-infected children under the base case PMTCT scenario. For these comparisons, a postnatal transmission rate assumed for women with a CD4 cell count of >350 mm^3^ was used (0.51%/month of breastfeeding) (16).

In the modelled scenarios, adherence to ARV prophylaxis was assumed to be 100%, given that the medicines had been disbursed. Since perfect adherence to ARV prophylaxis is rare, we also created alternative models for all the scenarios described above but assuming 50% adherence to ARV prophylaxis.

### Ethics

All participating women gave their informed consent. The home-based interviews with HIV-infected women after birth were carried out by an experienced midwife and the visits were carefully planned together with HDSS staff, midwives, and researchers not to reveal the women’s HIV status to anyone. Ethical approval for the study was obtained from Institutional Review Board, School of Public Health, Makerere University and the Uganda National Council of Science and Technology in 2008 (Ref. nr. HDREC, 052).

## Results

### HIV-infected children at birth and at 6 months with the base case PMTCT coverage

Given the HIV prevalence in the HDSS cohort of 3.5% and base case PMTCT coverage of ANC 96%, HIV testing 64% and ARV prophylaxis 83%, an estimated 13% of the children would be HIV-infected at birth in the Iganga and Mayuge districts (Table 2). At 6 months of age, this proportion would have increased to 16 or 20% depending on maternal CD4 cell count levels being more or equal/less than 350 mm^3^, respectively (Table 2). If comparing to a situation without any PMTCT programme, the base case PMTCT coverage did prevent 39% of child HIV infections at birth and 35 or 29% of the stipulated child HIV infections at 6 months of age, depending on if the CD4 count levels included was assumed to be above or below 350 mm^3^.

**Table 2:** Scenarios modelled for different coverage of PMTCT components, MTCT rates and number of children infected with HIV

		At birth^a^		At 6 months – CD 4 cell count >350/mm^3^ b		At 6 months – CD 4 cell count ≤350/mm^3^ b	
		
Scenario	Description	Number of children	MTCT rate (%)	Number of children	MTCT rate (%)	Number of children	MTCT rate (%)
1. Base case PMTCT coverage^c^	Number of HIV-infected children in the Iganga-Mayuge district population based on the empirical HDSS cohort data	240	13.4	284	15.8	369	20.5
2. 100% ANC	Base case scenario but 100% ANC attendance	234	13.0	278	15.5	363	20.2
3. 100% HIV testing	Base case scenario but 100% HIV testing	172	9.6	217	12.1	303	16.9
4. 100% ART	Base case scenario but ART provided to all women who received ARV prophylaxis	197	11.0	227	12.6	265	14.8
5. Optimal scenario^a^	A combination of Scenarios 2, 3, and 4	97	5.4	121	6.7	135	7.5
No PMTCT	No ARV prophylaxis given to HIV-infected women/babies	391	21.8	413	23.0	495	27.6

a Peripartum transmission rates for different ARV regimens: No ARV prophylaxis: 0.22, sd-NVP: 0.12, dual prophylaxis AZT and 3TC: 0.04, ART: 0.02

b Postpartum transmission rates per month of any breastfeeding (mixed or exclusive). Mother’s CD4 cell count ≤350 mm^3^: 0.0157, mother’s CD4 cell count >350 mm^3^: 0.0051, if mother receives ART: 0.002

c Base case PMTCT coverage obtained from the HDSS cohort: ANC 0.96, HIV testing 0.64, and ARV prophylaxis 0.83.

### MTCT rates for the modelled scenarios

The MTCT rates 6 months after delivery, for the different modelled scenarios range from 13% for the HDSS cohort, base case PMTCT coverage down to 5.4% for the optimal scenario (Table 2). The MTCT rate under the optimal scenario assuming a 100% coverage of ANC and HIV testing and a base case ARV prophylaxis coverage of 83% would result in a MTCT rate higher than the WHO target, which aims for MTCT rates below 5%.

### Assumed increased coverage of PMTCT components with 100% adherence to ARV prophylactic medicines

**Fig. 2:**
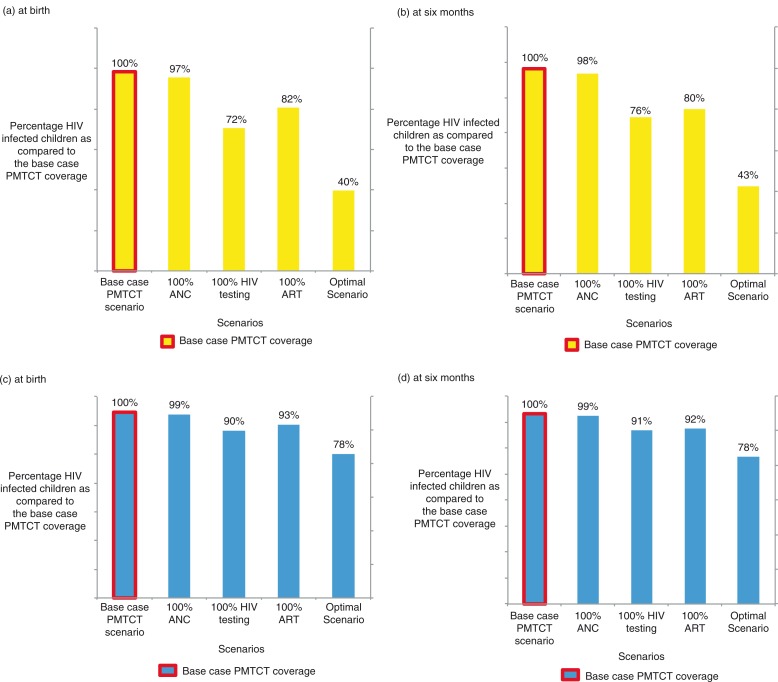
Estimated relative effects of an assumed increase in coverage of individual PMTCT programme components as compared to the base case PMTCT coverage. Assuming 100% (a, b) and 50% (c, d) adherence.

Since ANC attendance in this setting is as high as 96%, the effect on modelled MTCT of increasing ANC attendance to 100% is limited (Fig. 2a, b). Instead, the low coverage of HIV testing appears to be the major bottleneck for successful PMTCT in Iganga and Mayuge districts. An increase in HIV testing coverage to 100% among pregnant women attending ANC could reduce HIV infections among children by 28% at birth and by 24% at 6 months of age (Fig. 2a, b).

Increasing ART coverage to all women receiving ARV prophylaxis, while keeping all other components constant, would comparably only give an 18% reduction of infections at birth and 20% at 6 months.

The optimal scenario with 100% ANC attendance, 100% HIV testing, and providing ART to all the women receiving ARV prophylaxis (using the base case coverage of 83%) translates into a 60% decrease in infections at birth and a 57% decrease at 6 months compared to the number of children HIV infected with the base case coverage (Fig. 2a, b).

### Assumed increased coverage of PMTCT components with 50% adherence to ARV prophylactic medicines

Assuming a lower adherence level to ARV prophylactic medicines, of 50% but 100% HIV testing uptake, the number of HIV infections among children would be reduced by 10% at birth and 9% at 6 months compared to the estimated MTCT rate for the base case PMTCT scenario (Fig. 2c, d).

In this case, the optimal scenario would only provide a total reduction of 22% of infections at birth and at 6 months compared to number of children estimated to be HIV infected with the base case PMTCT coverage (Fig. 2c, d).

## Discussion

The study shows that in this rural Ugandan context with a resource-poor health system, the base case PMTCT coverage decreased the modelled MTCT rates from 22 to 13% at birth and from 23 to 16% at 6 months, as compared to a modelled scenario of no access to PMTCT services. However, these MTCT rates are still far from the MTCT-elimination targets of reducing global child HIV infections by 2015 (17).

Our study highlights the fact that health system interventions aimed to increase the uptake of HIV testing, and/or increased access to ART, could significantly reduce MTCT of HIV. The possibility to reach considerably better results in PMTCT by increasing HIV testing is especially interesting. We found in a previous study from the same area that the main determinant of being HIV tested among women seeking ANC was that the facility could offer onsite HIV testing since the referral from rural peripheral ANC clinics for HIV testing did not work (6). Hence, our results suggest that effectiveness of PMTCT programme could improve significantly by increasing the coverage of HIV testing, preferably by upgrading all peripheral ANC facilities to supply rapid HIV testing. The challenges associated with large-scale implementation of PMTCT programmes have generally been underestimated, partly because of the assumption that it may be linked to an already established intervention with high coverage, that is, ANC (18). However, our study emphasises the complexity of the PMTCT programme, and that the effectiveness of the programme is dependent on the uptake of each of the sequential PMTCT programme components.

Almost all (96%) pregnant women living with HIV had been in contact with the health care system through ANC, and should have been enrolled into the PMTCT programme according to the prevailing guidelines (10). Thus, the failure of this rural Ugandan health system to motivate women to test for HIV or to refer them for an HIV test reflects missed opportunities to prevent infant HIV infection and associated morbidity and mortality.

Our data showed that among the HIV-exposed children, an estimated 13% would be infected at birth. This could be compared to the modelling by Barker et al. showing that 80% ANC attendance, 80% HIV testing, and 80% access to ARV prophylaxis would translate into a 16% versus 12% MTCT if women with CD4 >350 received sd-NVP versus AZT/3TC (7). Our results support, and complement, the Barker study since we used population-based cohort data, and included all PMTCT programme components, including the crucial step of infant feeding practices. However, we reach similar conclusions: the urgent need for health system strengthening and making known efficient PMTCT interventions more effective in order to reduce the MTCT in sub-Saharan Africa. In addition, we also point to the significant effects on MTCT by increasing HIV testing in this specific context.

A well-functioning health system is crucial to deliver the sequential interventions that build the PMTCT programme (6, 14, 18). A Rwandan study demonstrated that women attending ANC clinics where PMTCT and ART had been integrated were almost twice as likely to enrol in ART care as compared to women attending stand-alone clinics for ANC (19). The authors of this publication attribute this to comprehensive support to HIV-infected pregnant women, and stress that integration of HIV care is crucial (19).

However, integration of care requires not only support from donors and policymakers but also local managerial resources, a functioning infrastructure for basic laboratory analysis, available ARVs, available and motivated service providers to adequately train all ANC staff to handle or refer HIV-infected women both at primary and secondary level care. Hence, translating international and national policies into practice is associated with challenges in a context such as rural Uganda (6, 20, 21). In addition to health systems factors influencing access to PMTCT, our earlier work highlights the importance of stigma, poor female decision-making power, and lack of knowledge about HIV and PMTCT among pregnant women in this Ugandan setting (6). We therefore suggest that further actions to increase the number of women tested for HIV at ANC, and also provided the result, should be built on locally grounded efforts where a community perspective is essential. Increased awareness of PMTCT at community level, and interventions aimed at reducing HIV-related stigma would make it easier for ANC staff to motivate and refer women for HIV testing and PMTCT (6, 20, 21).

The ‘100% HIV testing’ scenario reduced infant HIV infections by 28% at birth and by 24% at 6 months, as compared to the base case scenario. These results highlight the necessity of increasing capacity for HIV testing at ANC facilities but also the need to implement the newest PMTCT guidelines from the WHO, stating that all HIV-infected mothers should receive ART at least throughout the breastfeeding period to reduce MTCT of HIV (option B). The interventions needed to increase the effectiveness of PMTCT programmes differ between settings and context-specific bottlenecks, such as poor ANC attendance, must be overcome to increase PMTCT effectiveness (12). Our results further show that uptake and adherence to ART, especially throughout the breastfeeding period, is crucial to decrease the MTCT. If all the women who received ARV prophylaxis (i.e. 83% of the HIV-infected women) would receive ART throughout the breastfeeding period, MTCT rates could be reduced to 6.7 or 7.5% depending on maternal immune status while breastfeeding. Thus, support to both midwives at ANC and pregnant women to get a solid understanding of the importance of taking medicines as prescribed, and following infant-feeding advise, is equally necessary.

### Methodological considerations

By visiting all the 12,000 households twice during the study period, we hope to have minimised the risk of missed pregnancies. However, if pregnant women with HIV were more likely to avoid the regular HDSS data collection, MTCT rates have been underestimated in this study. Studies have shown that women, who think they are at higher risk of HIV infection or know that they are HIV infected, may be less likely to go through HIV testing at ANC due to stigma (8).

A limitation is the fact that the analysis is based on modelling, where we use several assumptions and not real-life data. The results are therefore a simplification of a more complex reality. For example, we have assumed that if in the modelling increasing the coverage of one PMTCT component, all other components would be constant. This might actually not be the case; instead the components might be interrelated. Another limitation that also has to do with the modelling is that we, due to practical reasons, have ignored whether the scaling up of PMTCT components is actually feasible within this health system.

Open-ended questions on PMTCT adherence to women enrolled in the Iganga-Mayuge PMTCT programme revealed that our assumption of 100% adherence to any ARV regimen was unrealistically optimistic, therefore we also modelled scenarios assuming 50% adherence. A study from Zambia found that only 68% of women that had received the simplest ARV prophylactic regimen, sd-NVP, had actually taken it as prescribed (22). A 6-month period of breastfeeding was chosen but we acknowledge that this period is usually much longer in Uganda.

## Conclusions

In a weak health system such as in Uganda, identifying bottlenecks impairing the continuum of care is critical. Without specific analyses of the uptake of the different steps in the treatment cascade, effectiveness of a complex intervention such as PMTCT remains unknown, and to strategically target the limited resources available is not possible.

In this setting, insufficient access to HIV testing was identified to be the main bottleneck to prevent MTCT. The number of children being HIV infected could be reduced significantly if all ANC facilities could offer testing for HIV to pregnant women.

Finally, the study emphasises the benefits of providing ART to pregnant women and to support women in adhering to ART throughout the breastfeeding period, a PMTCT regimen that is currently being rolled out in Uganda.

## Authors’s contributions

All authors have read and agreed on the submitted manuscript. EL, GP, GT, AET, and AME designed the study; EL, RB, and EG collected the data;, EL, MS, and AET analysed the data and drafted the manuscript; EL, EG, GP, GT, AME, AET, and RB interpreted the results. All authors gave critical input on the manuscript.
